# Modulation of the Lipid Profile of Reconstructed Skin Substitutes after Essential Fatty Acid Supplementation Affects Testosterone Permeability

**DOI:** 10.3390/cells8101142

**Published:** 2019-09-25

**Authors:** Mélissa Simard, Pierre Julien, Julie Fradette, Roxane Pouliot

**Affiliations:** 1 Centre de Recherche en Organogénèse Expérimentale de l’Université Laval/LOEX, Québec, QC G1J 1Z4, Canada; melissa.simard.6@ulaval.ca (M.S.); Julie.Fradette@fmed.ulaval.ca (J.F.); 2 Axe Médecine Régénératrice, Centre de Recherche du CHU de Québec–Université Laval, Québec, QC G1J 1Z4, Canada; 3 Faculté de Pharmacie de l’Université Laval, Québec, QC G1V 0A6, Canada; 4 Axe d’Endocrinologie et de Néphrologie, Centre de Recherche du CHU de Québec–Université Laval, Québec, QC G1V 4G2, Canada; pierre.julien@crchudequebec.ulaval.ca; 5 Département de Chirurgie de l’Université Laval, Québec, QC G1V 0A6, Canada

**Keywords:** skin substitutes, skin barrier function, polyunsaturated fatty acids, tissue engineering, lipidomics

## Abstract

Skin models with efficient skin barrier function are required for percutaneous absorption studies. The contribution of media supplementation with n-3 and n-6 polyunsaturated fatty acids (PUFAs) to the development of the skin barrier function of in vitro skin models remains incompletely understood. To investigate whether PUFAs, alpha-linolenic acid (ALA, n-3 PUFA) and linoleic acid (LA, n-6 PUFA), could enhance the impermeability of a three-dimensional reconstructed human skin model, skin substitutes were produced according to the self-assembly method using culture media supplemented with either 10 μM ALA or 10 μM LA. The impact of PUFAs on skin permeability was studied by using a Franz cell diffusion system to assess the percutaneous absorption of testosterone and benzoic acid. Our findings showed that ALA supplementation induced a decrease in the absorption of testosterone, while LA supplementation did not significantly influence the penetration of testosterone and benzoic acid under present experimental conditions. Both ALA and LA were incorporated into phospholipids of the skin substitutes, resulting in an increase in n-3 total PUFAs or n-6 total PUFAs. Collectively, these results revealed the under-estimated impact of n-3 PUFA supplementation as well as the importance of the n-6 to n-3 ratio on the formation of the skin barrier of in vitro reconstructed human skin models.

## 1. Introduction

The primary role of the epidermis is to protect the organism from environmental damage and prevent water loss. Epidermal keratinocytes proliferate and then differentiate in a perfectly orchestrated process during which both lipid and protein expression profiles are modulated, resulting in the formation of a semi-permeable layer, the stratum corneum [1]. This outermost layer of the skin is composed of completely differentiated keratinocytes called corneocytes embedded in a lipid matrix. The skin is characterized by an active lipid metabolism, which contributes not only to the formation and the maintenance of the skin barrier function but also plays an important role in the membrane structure as well as in cell function [2,3]. During cornification, plasma membrane phospholipids (PLs) are hydrolyzed and fatty acids (FAs) are used to synthesize other lipids such as triglycerides (TGs), ceramides (CERs) and sterols [4,5]. This process leads to the formation of the lipid matrix, which is composed of 45% CERs, 35% cholesterol and 15% free FAs [6].

Changes in the proportions and organization of the lipid matrix are known to affect skin permeability [7,8]. Polyunsaturated fatty acids (PUFAs), such as α-linolenic acid (ALA), an omega 3 (n-3) PUFA, and linoleic acid (LA), an omega 6 (n-6) PUFA, are important components for skin barrier homeostasis [2,9]. The importance of LA in the skin was first reported by Burr and Burr in 1929 [10]. They showed that n-6 deficiency in rodents resulted in scaly skin associated with barrier function impairment. The permeability of the rodent skin was then restored after dietary supplementation with LA. These studies revealed that LA and ALA cannot be synthesized by the organism de novo, which is why they are described as essential fatty acids (EFAs). Consequently, EFAs are common phospholipid membrane constituents and their relative proportions vary depending on their acquirement through dietary sources [11]. Further studies found that esterified ω-hydroxy-ceramide (CER[EOS]) is crucial for the organization of the stratum corneum lipid matrix [12,13,14]. In fact, the ω-esterified LA moiety from CER[EOS] is hydrolyzed so that it can be covalently bound to involucrin, a protein of the cornified envelope, allowing the scaffolding of other lipids of the matrix [15,16]. When LA is absent, it is replaced by oleic acid (OA), which causes the disorganization of the matrix [7]. In addition, ALA and LA enter parallel pathways where they are converted into arachidonic acid (AA) and eicosapentaenoic acid (EPA) respectively, and can be further metabolized into bioactive lipids [17,18]. These bioactive lipids are important for skin health as they regulate several key metabolic functions involved in the process of the stratum corneum formation that are not yet fully understood [19,20].

In the past decade, in vitro skin models have been useful tools for the study of processes involved in the regulation of keratinocyte proliferation and differentiation, as well as for toxicological testing of skin products [21,22]. Tissue-engineered skin models are often produced under PUFA-deficient culture conditions [23]. As a consequence, they present alterations in their epidermal lipid content such as different proportions of their epidermal CER classes [24]. The skin barrier function of in vitro skin models also exhibits a higher permeability compared to native human skin [25,26,27]. Previous studies using n-6 PUFAs showed that the fatty acid composition of culture media could have a significant impact on the phospholipid content of keratinocytes cultured as monolayers [28,29,30], on the epidermal lipid content of reconstructed epidermis cultured on a de-epidermized dermis [31], and on the epidermal lipid content of skin substitutes produced with a collagen matrix [32,33]. However, no study has investigated whether this modulation of the epidermal lipid composition affects the barrier function of the skin substitutes. Moreover, the effects of n-3 PUFAs on the barrier function of skin have raised attention lately, but still remain unclear [9,34]. In the current study, culture media were supplemented with either ALA or LA during the production of tissue-engineered human skin substitutes, to investigate the impact of PUFAs on the epidermal lipid composition and skin permeability. This study brings new insight into the importance of the n-6 to n-3 ratio in vitro for the establishment of skin barrier function.

## 2. Materials and Methods

### 2.1. Cell Culture

The institutional review board of the Université Laval approved the study and volunteers signed a consent form in accordance with the Helsinki declaration and the guidelines of the Research Ethics Committee of the CHU de Québec–Université Laval. The fibroblasts and keratinocytes were extracted from the breast reduction skin biopsies of three Caucasian women aged from 18–49 years old. Cells were extracted (cell populations: ♀18, ♀46, ♀49) according to the method based on thermolysin and trypsin digestions published previously [35] and cells were banked in liquid nitrogen. Fibroblasts (passage 5) were seeded at 1 × 10^4^ cells/cm^2^ in Dulbecco-Vogt modified Eagle’s medium (DMEM) supplemented with 10% of Fetal Calf Serum (FCS, Invitrogen, Burlington, ON Canada) with 0.06 mg/mL penicillin G (Sigma, Oakville, ON, Canada) and 25 μg/mL gentamicin (Schering, Pointe-Claire, QC, Canada). Primary keratinocytes (passage 3) were seeded at 1 × 10^4^ cells/cm^2^ on a feeder layer of irradiated S3T3 murine fibroblasts at 1 × 10^5^ cells/cm^2^ in DMEM mixed with Ham’s F12 (DMEMH) (3:1). The medium was supplemented with 5% Fetal Clone II serum (Calbiochem, EMD, ON, Canada), 5 μg/mL insulin (Sigma), 0.4 μg/mL hydrocortisone (Calbiochem, EDM Biosciences, Gibbstown, NI, USA), 10^−10^ M cholera toxin (MP Biomedicals, Montreal, QC, Canada), 10 ng/mL human epidermal growth factor (Austral Biological, San Ramon, CA, USA), 0.06 mg/mL penicillin (Sigma), 25 μg/mL gentamicin (Schering). Cells were incubated at 37 °C in an 8% CO_2_ atmosphere and media were changed three times per week.

### 2.2. Production of Tissue-Engineered Skin Substitutes

Skin substitutes were produced according to the self-assembly method presented in the Supplementary Figure S1, which is a modified version of the method previously described by Jean et al. [36,37]. Briefly, the primary fibroblasts (passage 6) were seeded in 6-well plates (1.2 × 10^5^ cells per well) and cultured in DMEM supplemented with 50 μg/mL ascorbic acid (Sigma, Mississauga, ON, Canada) for 3 weeks. During this time, the fibroblasts produced their own extracellular matrix, which lead to the formation of manipulatable dermal sheets. Two sheets were superimposed to form the dermal equivalent, which was incubated for two additional days in a 100 mm Petri dish. Primary keratinocytes (passage 2) were then seeded on the dermal equivalent (1.2 × 10^6^ cells per dermal equivalent) and cultured for one week in DMEMH. The substitutes were raised to the air-liquid interface and cultured for 21 days to allow the complete differentiation of the reconstructed epidermis. After a total of 56 days of culture, biopsies of the skin substitutes were analyzed.

Culture media were changed three times per week. Alpha-linolenic and linoleic acids were purchased from Sigma (Mississauga, ON, Canada) and dissolved in 99% EtOH (Greenfield Global, Brampton, ON, Canada). EFA stock solutions were added to the culture media at a final concentration of 10 μM during the 56 days of culture. EFAs were incorporated directly into the FCS, which contains abundant bovine serum albumin, during the preparation of the culture media to increase their solubility. The same amount of 99% EtOH was added to the FCS for Substitute.

### 2.3. Histologic and Morphometric Analyses

Biopsies were fixed in Histochoice solution and embedded in paraffin. Five-micrometer-thick transverse tissue sections were cut, and Masson’s trichrome staining was performed. Two substitutes for each of the 3 donors were analyzed (*n* = 6). The thickness of the dermis and living epidermal layers was measured on the stained sections with the Image J software. Ten measurements in 3 different sections of each biopsy were performed (*n* = 18 for each condition).

### 2.4. Percutaneous Absorption

Percutaneous absorption was performed using the standard Franz diffusion cell technique, as described by Franz [38,39]. Experiments were carried out in accordance with Organization for Economic Cooperation and Development (OECD) guidelines. Briefly, the reconstructed tissues were placed between the receptor and the donor compartments of the diffusion cells (5 mL volume, 0.63 cm^2^ surface area; Crown Glass, Somerville, NJ, USA). The receptor compartment was filled with a 0.1 M phosphate buffered saline (PBS) solution at pH 7.4. For the testosterone assay, 5% bovine serum albumin was added to the PBS solution. The receptor compartment was maintained at 37 °C, keeping the skin temperature approximately at 32 °C. Testosterone and benzoic acid (Sigma, ON, Canada) were applied to the substitutes at a concentration of 4 mg/mL ethanol/water (1:1, *v*/*v*), corresponding to 400 μg in 100 μL for testosterone and 800 μg in 200 μL for benzoic acid, per substitute. The total exposure time was 24 h, during which samples were collected with a 5 mL syringe lengthened with a catheter (3 ½ FR Tom Cat Length 4 ½) at various time points (1, 2, 3, 4, 6, 8, 24 h post-dosing). Samples were conserved at 4 °C until ultra performance liquid chromatography (UPLC) quantification.

Samples were assayed by an in-house developed UPLC-UV method using a Waters Acquity UPLC system with a Waters photodiode array (PDA) detector and a thermostated autoinjector (Acquity UPLC H-Class System, Waters, Mississauga, ON, Canada). Testosterone (eluent: 50% water/50% ACN, flow: 0.6 mL/min, 248 nm) and benzoic acid (eluent: 80% water/20% ACN, flow: 0.6 mL/min, 230 nm) were separated on a BEH C_18_, Waters column (50 mm × 2.1 mm, 1.7 μm, ON, Canada) kept at 40 °C. The mobile phase was an acetonitrile/water solution buffered with a 0.1% trifluoroacetic acid. The injection volume was 5 μL. Under these conditions, testosterone was eluted at approximately 1.50 min and benzoic acid (BA) at 1.1 min. Data collection and peak integration were performed using the Empower 2 software (Waters, Mississauga, ON, Canada).

### 2.5. Gas Chromatography

The epidermis of skin substitutes was mechanically separated from the dermis using forceps and scalpels and for human skin (6 Caucasian donors: 2 males and 4 females aged from 26–48 years old), the epidermis was peeled off using forceps after incubation in water at 60 °C for 1 min. Epidermal and dermal samples were quick-frozen in liquid nitrogen and conserved at −80 °C until analyzed. Extraction of the epidermal and dermal lipids was performed using a chloroform-methanol mixture (2:1, *vol/vol*) according to a modified Folch method [40]. Total PLs were separated by thin layer chromatography. A first migration was performed in isopropyl ether/acetic acid (96/4) until the middle of the plate was reached. A second migration was then performed in another chamber using petroleum ether/ethyl ether/acetic acid (90/10/1). Fatty acids of isolated PLs were methylated. Capillary gas chromatography with a HP5890 gas chromatograph (Hewlett-Packard, Toronto, ON, Canada) equipped with an HP-88 capillary column (100 mm × 0.25 mm internal diameter, 0.20 μm film thickness; Agilent Technologies, Santa Clara, CA, USA) coupled with a flame ionization detector was then used to obtain FA profiles, as described elsewhere [41]. The unsaturation index (UI) was calculated according to the formula [42]:

UI = ((%Monoenoic FA × 1) + (%Dienoic FA × 2) + (%Trienoic FA × 3) + (%Tetraenoic FA × 4) + (%Pentaenoic FA × 5) + (%Hexaenoic FA × 6))/%Saturated FA.(1)

### 2.6. Statistical Analysis

Data were analyzed using Student’s *t*-tests and expressed as means ± standard deviation for parametric variables, except when stated otherwise. Statistical analyses of FA lipid profiles were performed using analyses of variance (ANOVAs) with Tukey’s or Sidak’s post-hoc tests. Only values of *p* < 0.05 were considered significant. All calculations were performed with the Prism version 7 software (GraphPad Software, La Jolla, CA, USA).

## 3. Results

### 3.1. Morphology of the Skin Substitutes

**Figure 1:**
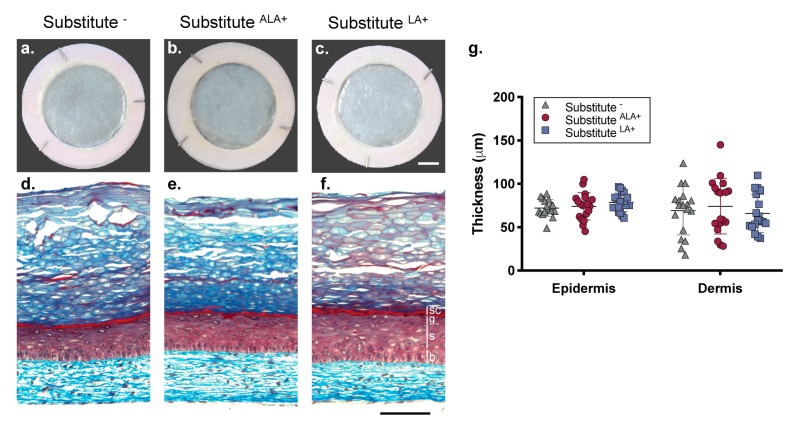
Impact of EFA supplementation on skin substitute cutaneous morphology. (**a–c**) Macroscopic aspect and (**d**–**f**) histological cross-section after Masson’s trichrome staining of the skin substitutes. Vertical bars indicate epidermal layers. b: basal layer, s: spinous layer, g: granular layer and sc: compact layers of the stratum corneum. Scale bars: (a–c) 1 cm, (d–f) 100 μm. (**g**) Epidermal and dermal thickness quantified from Masson’s trichrome staining. *N* = 18 (3 donors, 2 skin substitutes per donor, 3 measurements per skin substitute). One-way ANOVA followed by Tukey’s *post-hoc* test. *p* < 0.05 was considered statistically significant.

Skin substitutes were produced according to the self-assembly method of tissue engineering using culture media supplemented with 10 μM ALA (Substitute^ALA+^) or 10 μM LA (Substitute^LA+^) (see Supplementary Figure S1). The effect of several concentrations of LA on the morphology of the skin substitutes was assessed (5, 10, 20, 50, 75, 100, and 150 μM), leading to the selection of a concentration of 10 μM (see Supplementary Figure S2). Similar morphology was observed in the Substitute^−^, Substitute^ALA+^ and Substitute^LA+^ (Figure 1). The epidermis of the skin substitutes produced with or without the addition of EFAs showed a uniform morphology (Figure 1a–c). Masson’s trichrome staining of skin substitute cross-sections showed that the substitutes supplemented with both EFAs displayed an epidermis with a stratum corneum (Figure 1d–f). Since tissue thickness can affect skin permeability, measurements of the epidermal (section designed by the vertical bars in Figure 1) and dermal thickness of the skin substitutes were performed with Image J software (version 1.51s). The thickness of the Substitute^ALA+^ and Substitute^LA+^ were not significantly different from the Substitute^−^ (Figure 1g).

### 3.2. Effects of Essential Fatty Acids on the Percutaneous Absorption of Testosterone and Benzoic Acid Through Skin Substitutes

**Figure 2:**
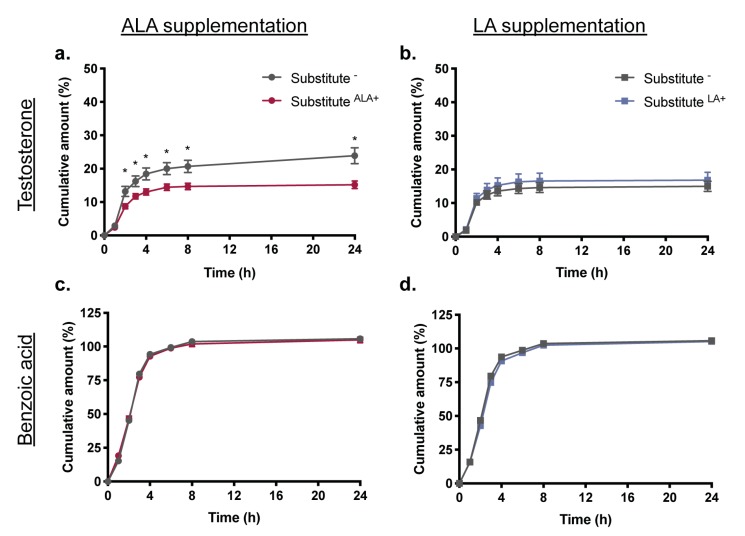
Skin substitute permeability to testosterone and benzoic acid. Influence of (**a**,**c**) ALA and (**b**,**d**) LA supplementation on the cumulative dose of (**a**,**b**) testosterone and (**c**,**d**) benzoic acid absorbed through the skin substitutes. Percutaneous absorption studies were performed on a Franz cells diffusion system. The dosing solutions were freshly prepared in ethanol/water (1:1), yielding a concentration of 4.0 mg/mL for each compound. Compounds were quantified with a Waters Acquity UPLC. Values are mean +/− standard error of the mean (SEM) (3 donors, 6 skin substitutes per donor), *p*-values were derived from Student’s *t*-tests. * *p* < 0.05.

To evaluate the impact of EFA supplementation on the barrier function of skin substitutes, percutaneous absorption studies of testosterone and benzoic acid, which are respectively lipophilic and hydrophilic, were performed using a Franz cell diffusion system. Interestingly, ALA supplementation of the culture conditions leads to decreased absorption of testosterone through the skin substitutes. Indeed, the cumulative amount of testosterone in the receptor fluid was significantly less for Substitute^ALA+^ than for the control Substitute^−^ at 2, 3, 4 and 6 h (Figure 2a). The maximum flux of testosterone at 2 h was also significantly less through the Substitute^ALA+^ (−1.7-fold) (Table 1). A decrease of maximum fluxes of testosterone was measured for Substitute^ALA+^ produced with cells from three donors resulting in 1.7, 1.9, and 1.45-fold decreases respectively, although a statistically significant difference was only reached with the second donor. In contrast, supplementation with LA did not affect the cumulative amount (Figure 2b) or the mean maximum fluxes of testosterone through the skin substitutes produced with cells from the three donors (*p* > 0.05) (Table 1). After 24 h, the receptor fluid of the Substitute^ALA+^ contained less testosterone than that of Substitute- (Substitute^ALA+^: 15.2 ± 1.1%, Substitute^−^: 23.9 ± 2.4%, *p* < 0.05) while the same amounts were collected for Substitute^LA+^ and Substitute- (Substitute^LA+^: 16.8 ± 2.4%, Substitute^−^: 15.0 ± 1.5%, *p* > 0.05) (see Supplementary Figure S3). Most of testosterone was found in the epidermis or in the lavage fractions. The total recovery of testosterone ranged between 82.7% (Substitute^ALA+^) and 107.5% (Substitute^LA+^). No significant differences were observed between the total recovery amounts of testosterone whether it be for Substitute^ALA+^, Substitute^LA+^ or their respective controls.

BA absorption through the skin substitutes was not significantly affected by supplementations with either EFAs under our experimental conditions. The amount of BA detected in the receptor fluid was not affected by supplementation with either ALA (Figure 2c) or LA (Figure 2d). However, observing the BA mean fluxes, which rapidly increased during the first 3 h (Table 1) and then decreased until 24 h (not shown), revealed that the maximum absorption rate at 3 h of BA through Substitute^ALA+^ was significantly less than for Substitute^−^ (−1.2-fold). No significant difference was observed for BA fluxes through Substitute^LA+^ (Table 1).

### 3.3. Fatty Acids Added to the Culture Media: Incorporation into the Epidermal and Dermal Phospholipids

**Figure 3:**
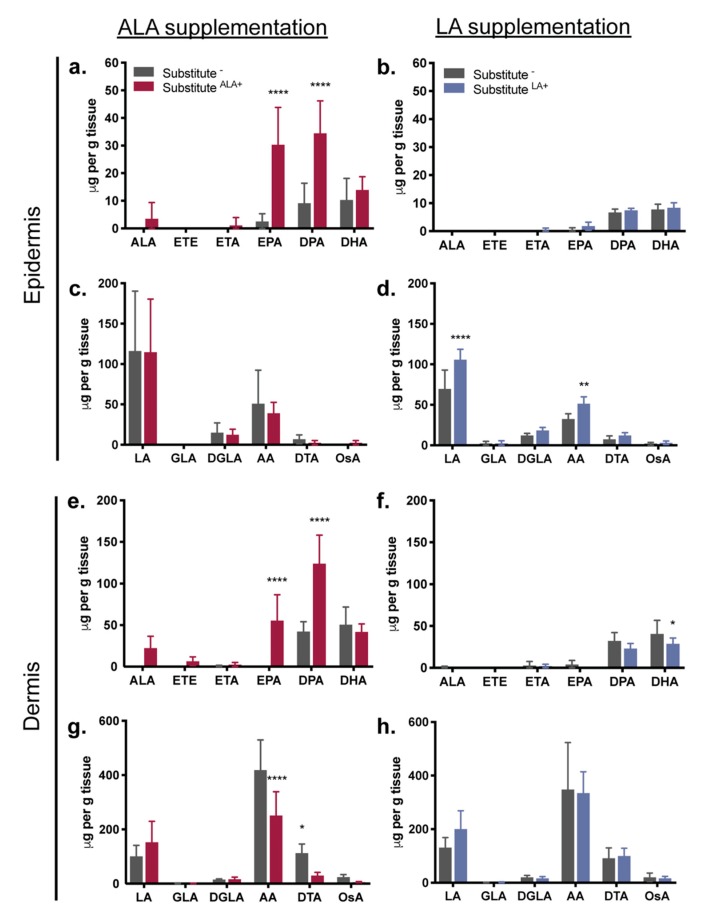
EFA incorporation in the epidermal and dermal phospholipid fraction of the skin substitutes. (**a**,**c**,**e**,**g**) ALA supplementation impact on (**a**,**e**) n-3 PUFAs and (**c**,**g**) n-6 PUFAs and (**b**,**d**,**f**,**h**) LA supplementation impact on (**b**,**f**) n-3 PUFAs and (**d**,**h**) n-6 PUFAs. PUFAs were quantified following gas chromatography analysis. Results are expressed as μg per g of tissue. Two-way ANOVA followed by Sidak’s *post-hoc* test. * *p* < 0.05; ** *p* < 0.01; *** *p* < 0.001; **** *p* < 0.0001. For skin substitutes: *n* = 6 (3 donors, 2 skin substitutes per donor). Abbreviations: AA, arachidonic acid; ALA, α-linolenic acid; DGLA, dihomo-γ-linolenic acid; DHA, docosahexaenoic acid; DPA, docosapentaenoic acid; DTA, docosatetraenoic acid; EPA, eicosapentaenoic acid; ETA, eicosatetraenoic acid; ETE, eicosatrienoic acid: GLA: γ-linolenic acid; LA, linoleic acid; OsA, osbond acid; PUFAs, polyunsaturated fatty acids.

To evaluate the EFA incorporation resulting from media supplementations, phospholipids were extracted from the skin substitutes and their FA profiles were analyzed by gas chromatography. It appeared that the added essential fatty acids (ALA and LA) were successfully taken up from the culture media and incorporated into the PLs of the epidermal and dermal cells (Figure 3). For ALA supplementation, although the amount of ALA did not significantly increase in the epidermal and dermal PL fractions, significant increases in the levels of ALA metabolites were found, thus suggesting that ALA was further metabolized into EPA (epidermis: 12-fold) and docosapentaenoic acid (DPA) (epidermis: 3.75-fold) (Figure 3a,e). Exogenous provision of ALA did not increase the level of docosahexaenoic acid (DHA) (*p* > 0.05) (Figure 3a,e). The ALA supplementation was associated with a reduction in dermal n-6 PUFA levels, more precisely less AA (-1.6-fold) and docosatetraenoic acid (DTA) (-3-fold) were found in Substitute^ALA+^ (Figure 3g).

For LA supplementation, a significant increase in LA (1.5-fold) was measured in the epidermal compartment of the Substitute^LA+^ as well as a significant increase in AA (1.6-fold), one of the LA metabolites (Figure 3d). Conversely, a higher level of LA (1.5-fold) was found in the dermal compartment, although it did not reach statistical significance (Figure 3h). The LA supplementation induced a decrease in DHA n-3 PUFA (-1.4-fold) in the Substitute^LA+^ dermis (Figure 3f). Detailed fatty acid concentrations expressed in μg/g of tissue and used to produce Figure 3 are also available in Supplementary Table S1.

### 3.4. The Proportion of n-3 and n-6 Fatty Acids Increases in Response to ALA and LA Supplementation, Causing Changes in n-3 to n-6 Ratios and Unsaturation Indexes

**Figure 4:**
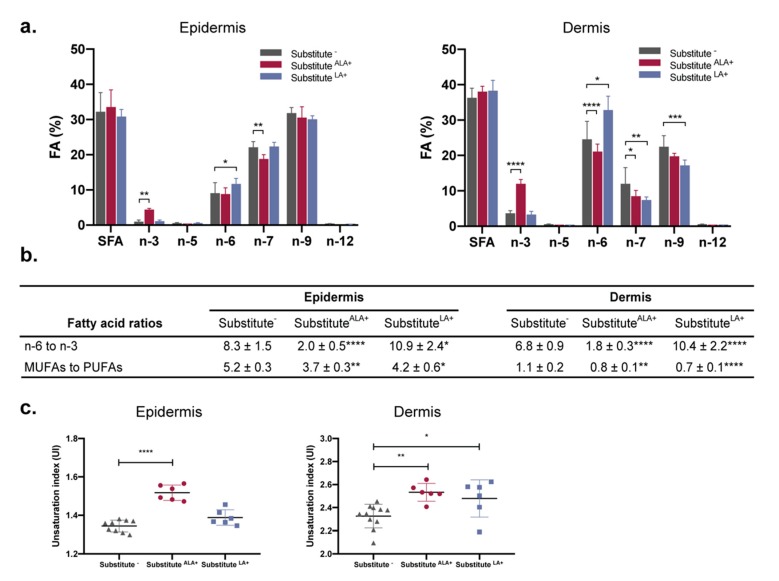
Proportions of FAs in the skin substitutes quantified by gas chromatography. (**a**) FA classes are shown as a percentage of total FAs detected in phospholipids of the epidermis or the dermis. (**b**) Phospholipid FA ratios in the epidermis or the dermis of the skin substitutes. (**c**) UI of the phospholipid FAs in the epidermis and the dermis. For Substitute^−^: *n* = 11 (3 donors, 3-4 skin substitutes per donor); for Substitute^ALA+^ and Substitute^LA+^: n = 6 (3 donors, 2 skin substitutes per donor). (**a**) Two-way ANOVA followed by Tukey’s *post-hoc* test. (**b**,**c**) One-way ANOVA followed by Tukey’s *post-hoc* test. * *p* < 0.05; ** *p* < 0.01; *** *p* < 0.001; **** *p* < 0.0001. Abbreviations: ALA, α-linolenic acid; FA, fatty acid; LA, linoleic acid; MUFAs, monounsaturated fatty acids; PUFAs, polyunsaturated fatty acids; SFA, saturated fatty acid; UI, unsaturation index.

The impact of FA incorporation on the global epidermal and dermal FA profiles of the skin substitutes was examined by observing the relative proportions of FA classes: saturated fatty acids (SFAs), monounsaturated fatty acids (MUFAs) (n-5, n-7 n-9 and n-12) and PUFAs (n-3 and n-6) (Figure 4a). A significant increase in the relative proportion of total PL n-3 FAs was found in both the epidermis (4.3-fold) and dermis (3.3-fold) after ALA supplementation (Figure 4a), which is in accordance with the increase of individual n-3 FAs (EPA and DPA) previously shown in Figure 3. The higher n-3 FA proportion was associated with a reduced n-7 FA proportion in the epidermis, as well as reduced n-6 and n-7 FA proportions in the dermis. The LA supplementation induced an increase in the proportion of total epidermal and dermal PL n-6 FAs (1.3-fold). Furthermore, proportions of n-7 and n-9 MUFAs were found to be significantly decreased in dermal PLs after supplementation with LA (Figure 4a).

The high level of n-3 PUFAs found in the Substitute^ALA+^ were sufficient to significantly decrease the ratio of n-6 to n-3 FAs in the epidermal (-4.2-fold) and dermal (-3.8-fold) PLs (Figure 4b). Inversely after LA supplementation, the same ratios were found to be increased in epidermal (1.3-fold) and dermal (1.5-fold) compartments (Figure 4b). After ALA and LA supplementation, the ratios of MUFAs to PUFAs were significantly decreased in both compartments (Figure 4b). The unsaturation index (UI), a calculation which is based on the amount and unsaturation numbers of FAs, is a parameter that reveals information about membrane biophysical properties and behavior. The UIs were higher in epidermal and dermal compartments after ALA supplementation as compared to the control, thus suggesting an increase in cell membrane fluidity (Figure 4c). However, after LA supplementation, the UI was found to be increased in dermal tissue only.

The phospholipid fatty acid characterization was also performed on human skin biopsies. Higher levels of PUFAs were found in skin biopsies than in reconstructed skin substitutes (Supplementary Figure S4).

## 4. Discussion

Many studies have shown the importance of n-6 PUFAs for the in vivo formation of the skin barrier [10,43,44]. Reconstructed skin models produced using classical culture media not enriched in exogenous lipids were proven relevant models for the study of the effects of PUFAs on the formation of the skin barrier, as they indeed displayed a PUFA-deficient lipid profile with lower concentrations of n-3 and n-6 FAs but greater amounts of MUFAs than found in normal human skin [23]. Our findings showed that skin substitutes produced with media supplemented with ALA were more impermeable to testosterone, while supplementation of the media with LA had no impact on the skin substitutes’ permeability for the molecules tested. Modulation of the skin substitutes’ permeability could be attributed to an enhanced n-3 total fatty acid presence in the skin substitutes. Taken together, these results highlight the importance of the balance between n-6 and n-3 PUFAs in the development of an efficient skin barrier.

Studies in rodents have shown that in contrast to n-6 PUFAs, n-3 PUFAs were not essential for obtaining an impermeable skin barrier [45,46]. To our knowledge, this current study is the first to show that n-3 supplementation can actually modulate the skin barrier function in an in vitro reconstructed human skin model, based on the observation that decreased testosterone absorption is indicative of improved barrier function. Surprisingly, supplementation with LA did not translate into a measurable difference in the percutaneous absorption as shown using the Franz cell diffusion system. This contrasts with the observations made in vivo in rodents for which enhanced skin permeability induced by a n-6 deficient diet could be restored to an impermeable barrier using dietary supplementation with LA [10,43].

**Figure 5:**
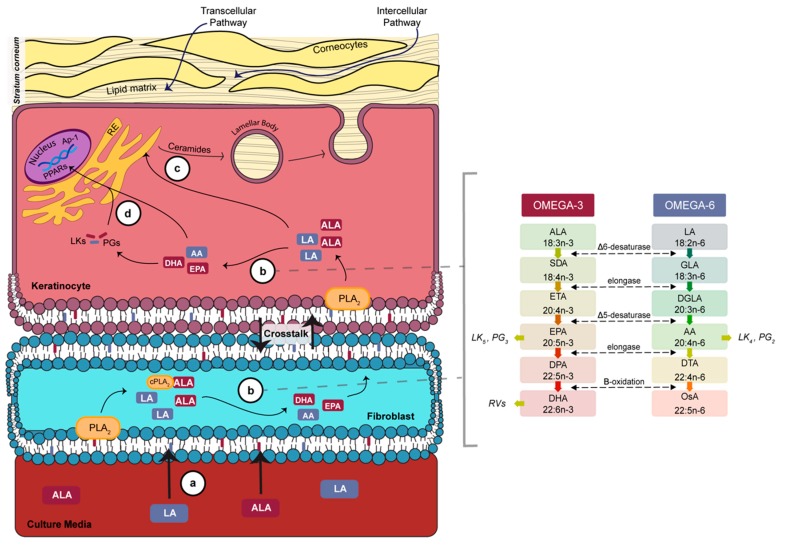
Schematic overview of n-3 and n-6 metabolism in the skin at the air-liquid interface. (**a**) The ALA and LA PUFAs added into the culture media are incorporated into the phospholipids of cells in contact with the media. During the air-liquid interface culture period, keratinocytes are no longer in contact with the culture media and PUFAs must be provided from the crosstalk with fibroblasts. (**b**) The PLA_2_ hydrolyzes PUFAs from the phospholipids. ALA and LA can be converted by a series of desaturation and elongation reactions in both fibroblasts and keratinocytes. (**c**) After phospholipid hydrolysis, PUFAs can also be brought to the endoplasmic reticulum where they are transformed into more complex lipids such as ceramides. Lipids are stored in lamellar bodies, which then merge with the cell membrane, releasing their content and thus forming the intercellular lipid matrix. (**d**) PUFAs such as EPA and DHA are activators of PPARs, which regulate keratinocyte differentiation. Abbreviations: AA, arachidonic acid; ALA, α-linolenic acid; Ap-1, activator protein 1; COX, cyclooxygenase; cPLA_2_, cytosolic phospholipase A2; DGLA, dihomo-γ-linolenic acid; DHA, docosahexaenoic acid; DPA, docosapentaenoic acid; DTA, docosatetraenoic acid; EPA, eicosapentaenoic acid; ETA, eicosatetraenoic acid; GLA, γ-linolenic acid; LA, linoleic acid; LK, leukotriene; LOX, Lipoxygenase; OsA, osbond acid; PG, prostaglandin; PLA_2_, phospholipase A2; PPAR, peroxisome proliferator-activated receptor; PUFAs, polyunsaturated fatty acids; SDA, stearidonic acid.

Testosterone and BA, respectively lipophilic and hydrophilic compounds, are substances recommended by the OECD guidelines and have therefore been extensively studied in the evaluation of percutaneous absorption [26,47,48]. According to their physico-chemical properties, it is likely that lipophilic molecules predominantly penetrate the stratum corneum through the intercellular pathway (lipid matrix) while on the contrary, hydrophilic molecules penetrate through the transcellular pathway (protein-filled cells) as illustrated in Figure 5 [49,50,51]. Based on these concepts, since ALA supplementation modulates lipophilic absorption, the impact of ALA on the barrier function may be more significantly caused by a modulation of the lipid content of the skin substitute, which is in agreement with the phospholipid fatty acid profiles reported in the present study. In fact, our study is the first to report the proper incorporation of n-3 PUFAs into the epithelial cells of a reconstructed model following n-3 PUFA supplementation. However, whether mechanisms of absorption can be completely dominated by one of the two pathways or are the result of a partitioning between each route remains unclear, thus an impact on the intercellular pathway must not totally be excluded [50]. Furthermore, since LA supplementation had no impact on percutaneous absorption values in the present model, it would suggest that LA may not have a significant impact on the epidermal lipid content of the skin substitutes. However, an increase in n-6 PUFAs was interestingly observed following LA supplementation, which is in agreement with other studies using different skin models [28,31,32].

Analyses of the epidermal and dermal phospholipid profiles are providing us with new concepts about the percutaneous absorption results obtained after PUFA supplementation. Moreover, studying both the lipid content of the epidermis and the dermis allows us to appreciate the distribution of the incorporated EFAs between the two skin layers. ALA and LA are competitive molecules as they are using the same enzymatic pathways to be converted into their metabolites [52]. Consequently, high levels of n-3 PUFAs promote their metabolization, to the detriment of n-6 PUFA metabolization [53]. In the present study, the incorporation of ALA into the skin substitute cells resulted in an increase in total n-3 FAs in both the dermal and epidermal compartments, with EPA and DPA being the predominant enhanced FAs. Interestingly, the exogenous provision of ALA did not increase the level of DHA, confirming the slow rate of conversion of DPA to DHA, an observation previously reported in nutritional studies [54,55], and/or the retroconversion of DHA to EPA [56]. Furthermore, ALA incorporation also resulted in a decrease in AA in the dermis, showing decreased n-6 metabolization. On the contrary, LA supplementation resulted in higher levels of n-6 PUFAs (LA and AA) in the epidermis as well as decreased levels of DHA (n-3) in the dermis. EFA supplementation of the culture media induced different responses in the lipid profile of FA phospholipids between the epidermis and the dermis, indicating skin layer differences in lipid metabolism, which could be attributed to differences in enzyme expression between compartments.

Lower n-6 to n-3 ratios, such as the ratios found in the present study after ALA supplementation, may lead to decreased metabolization of LA into AA and thus, a larger pool of LA available for CER[EOS] synthesis. In vivo studies using an atopic dermatitis rat model showed that EPA ethyl ester could enhance the production of bound CERs [34]. On the other hand, the added LA may be actively converted into AA instead of being incorporated into CER[EOS]. It has been shown by injecting either LA or AA in rats with a n-6-deficient diet that the injection of AA may reduce excessive scaling of the skin, but only LA restored the skin barrier function [43]. Further investigation of the FA composition of CERs would be of great interest to determine if the CER composition of skin substitutes is affected by EFA supplementation.

More detailed analyses of the lipid profiles suggest that PUFA supplementation could affect the membrane fluidity of epithelial cells. Supplementation with both EFAs tends to diminish the PUFA-deficient profile, showing higher PUFA levels and lower MUFA levels, which is in agreement with a previous study [31]. A smaller MUFA to PUFA ratio in PLs is associated with higher cellular membrane fluidity [57]. A study on keratinocytes cultured with either EPA or AA showed that PUFA incorporation into the PLs enhanced cell membrane fluidity [58]. In the present study, only ALA supplementation was significantly correlated with an increased UI in the epidermis, which suggests that n-3 PUFAs would have a bigger impact on the cell membrane fluidity than n-6 PUFAs. A higher impact on the UI of n-3 PUFAs could be attributed to further metabolization of n-3 PUFAs rather than of n-6 PUFAs in the skin. As observed in our study, the increase in EPA was related to an important increase in DPA, while no n-6 PUFAs other than AA increased in keratinocyte PLs. We hypothesize that a higher fluidity of the keratinocyte membrane could influence the formation of the stratum corneum and thus could influence skin permeability. Indeed, the differentiation of keratinocytes, resulting in the formation of the *stratum corneum*, involves a major reorganization of the transmembrane proteins, such as transglutaminase and involucrin [59,60], that could be facilitated by higher membrane fluidity. Moreover, the extrusion of lamellar bodies in the uppermost layer of the epidermis is a crucial step in the formation of the functional barrier. This process may also be influenced by cell membrane fluidity. It is well known that other process also involving invagination or fusion of cell membranes, such as phagocytosis, are impacted by the PUFA composition of the membrane [61].

The incorporation of n-3 PUFAs into the skin substitute cells could have influenced the percutaneous absorption of testosterone through several other mechanisms. N-3 fatty acids, especially DHA, are well-known activators of the peroxisome proliferator-activated receptors (PPARs), which regulate lipid metabolism, keratinocyte differentiation and the expression of the cornified envelope proteins [62,63,64]. It was shown in a mouse model that the activation of PPARs with an agonist accelerates the repair of the barrier function after acute disruption [65]. The n-3 PUFAs are also important for skin homeostasis, as they can be transformed into bioactive lipids [18,66]. Further studies will be needed to investigate other mechanisms by which the modulation of the PL FA profiles following long-term ALA supplementation could be responsible for the decreased testosterone absorption.

## 5. Conclusions

In summary, we discovered that the permeability of a reconstructed human skin model is affected by lipids from the culture media. The FA composition of the culture media during the production of skin substitutes should be further investigated as the proportion of the different FA classes in the media is important for the formation of the skin barrier. Moreover, n-3 PUFA levels can influence skin permeability as observed with the decreased absorption of testosterone after ALA supplementation. Enhanced n-3 PUFA levels in PLs could promote the formation of the skin barrier by reducing the conversion of LA to AA and by regulating cell membrane fluidity. To conclude, this study highlights the importance of the n-6 to n-3 ratio for the integrity of the skin barrier function of tissue-engineered human skin substitutes.

## Figures and Tables

**Table 1:** Mean maximum fluxes of testosterone (2 h) and benzoic acid (3 h) through the skin substitutes.

Molecules	Donors	Substitute^−^		Substitute^ALA+^		*p*-Value	Substitute^−^		Substitute^LA+^		*p*-Value
		Flux (μg/cm^2^/h)	*n*	Flux (μg/cm^2^/h)	*n*		Flux (μg/cm^2^/h)	*n*	Flux (μg/cm^2^/h)	*n*	
**Testosterone**											
	1	62.8 ± 7.6	7	37.6 ± 12.6	7	NS	35.1 ± 2.9	8	32.6 ± 3.7	8	NS
	2	94.9 ± 7.2	6	49.4 ± 7.3	6	0.0013	94.9 ± 7.2	6	101.5 ± 13.7	6	NS
	3	79.7 ± 29.4	6	54.9 ± 4.0	6	NS	37.1 ± 3.7	7	37.3 ± 5.5	7	NS
	*Mean*	78.3 ± 9.9	19	46.8 ± 5.3	19	0.008	52.9 ± 6.4	21	48.8 ± 9.7	21	NS
**Benzoic Acid**											
	1	456.2 ± 25.7	7	369.8 ± 11.2	7	0.0095	456.2 ± 25.7	7	396.3 ± 11.0	7	NS
	2	417.7 ± 3.3	6	389.2 ± 9.1	4	0.0087	417.7 ± 3.3	6	399.3 ± 4.3	5	0.0072
	3	437.9 ± 7.7	5	444.3 ± 11.9	5	NS	380.2 ± 20.8	7	410.7 ± 16.5	7	NS
	*Mean*	438.3 ± 10.6	18	397.9 ± 10.4	16	0.0105	418.0 ± 13.2	20	402.4 ± 7.2	19	NS

All data are mean ± SEM. Statistical analyses were performed with Student’s *t*-test. Abbreviation: SEM, standard error of the mean.

## Supplementary Materials

The Supplementary Materials are available online at https://www.mdpi.com/2073-4409/8/10/1142/s1.

## References

[1] Madison K.C. (2003). Barrier function of the skin: “la raison d’etre” of the epidermis. J. Investig. Dermatol..

[2] Feingold K.R., Elias P.M. (2014). Role of lipids in the formation and maintenance of the cutaneous permeability barrier. Biochim. Biophys. Acta.

[3] Kruse V., Neess D., Faergeman N.J. (2017). The Significance of Epidermal Lipid Metabolism in Whole-Body Physiology. Trends Endocrinol. Metab..

[4] Meckfessel M.H., Brandt S. (2014). The structure, function, and importance of ceramides in skin and their use as therapeutic agents in skin-care products. J. Am. Acad. Dermatol..

[5] Freinkel R.K., Traczyk T.N. (1977). Flux of fatty acids during epidermal differentiation. J. Investig. Dermatol..

[6] Yardley H.J., Summerly R. (1981). Lipid composition and metabolism in normal and diseased epidermis. Pharmacol. Ther..

[7] Bouwstra J.A., Ponec M. (2006). The skin barrier in healthy and diseased state. Biochim. Biophys. Acta.

[8] Van Smeden J., Janssens M., Gooris G.S., Bouwstra J.A. (2014). The important role of stratum corneum lipids for the cutaneous barrier function. Biochim. Biophys. Acta.

[9] Kendall A.C., Kiezel-Tsugunova M., Brownbridge L.C., Harwood J.L., Nicolaou A. (2017). Lipid functions in skin: Differential effects of n-3 polyunsaturated fatty acids on cutaneous ceramides, in a human skin organ culture model. Biochim. Biophys. Acta.

[10] Burr G.O., Burr M.M. (1929). A new deficiency disease produced by the rigid exclusion of fat from the diet. J. Biol. Chem..

[11] Russo G.L. (2009). Dietary n-6 and n-3 polyunsaturated fatty acids: From biochemistry to clinical implications in cardiovascular prevention. Biochem. Pharm..

[12] Bouwstra J.A., Gooris G.S., Dubbelaar F.E., Weerheim A.M., Ijzerman A.P., Ponec M. (1998). Role of ceramide 1 in the molecular organization of the stratum corneum lipids. J. Lipid Res..

[13] Melton J.L., Wertz P.W., Swartzendruber D.C., Downing D.T. (1987). Effects of essential fatty acid deficiency on epidermal O-acylsphingolipids and transepidermal water loss in young pigs. Biochim. Biophys. Acta.

[14] Mojumdar E.H., Gooris G.S., Groen D., Barlow D.J., Lawrence M.J., Deme B., Bouwstra J.A. (2016). Stratum corneum lipid matrix: Location of acyl ceramide and cholesterol in the unit cell of the long periodicity phase. Biochim. Biophys. Acta.

[15] Behne M., Uchida Y., Seki T., de Montellano P.O., Elias P.M., Holleran W.M. (2000). Omega-hydroxyceramides are required for corneocyte lipid envelope (CLE) formation and normal epidermal permeability barrier function. J. Investig. Dermatol..

[16] Popa I., Watson A.L., Solgadi A., Butowski C., Allaway D., Portoukalian J. (2018). Linoleate-enriched diet increases both linoleic acid esterified to omega hydroxy very long chain fatty acids and free ceramides of canine stratum corneum without effect on protein-bound ceramides and skin barrier function. Arch. Dermatol. Res..

[17] Nicolaou A. (2013). Eicosanoids in skin inflammation. Prostaglandins Leukot. Essent. Fat. Acids.

[18] Kendall A.C., Pilkington S.M., Massey K.A., Sassano G., Rhodes L.E., Nicolaou A. (2015). Distribution of bioactive lipid mediators in human skin. J. Investig. Dermatol..

[19] Mayser P., Grimm H., Grimminger F. (2002). n-3 fatty acids in psoriasis. Br. J. Nutr..

[20] Clark C.C.T., Taghizadeh M., Nahavandi M., Jafarnejad S. (2019). Efficacy of omega-3 supplementation in patients with psoriasis: A meta-analysis of randomized controlled trials. Clin. Rheumatol..

[21] Netzlaff F., Lehr C.M., Wertz P.W., Schaefer U.F. (2005). The human epidermis models EpiSkin, SkinEthic and EpiDerm: An evaluation of morphology and their suitability for testing phototoxicity, irritancy, corrosivity, and substance transport. Eur. J. Pharm. Biopharm..

[22] Almeida A., Sarmento B., Rodrigues F. (2017). Insights on in vitro models for safety and toxicity assessment of cosmetic ingredients. Int. J. Pharm..

[23] Marcelo C.L., Duell E.A., Rhodes L.M., Dunham W.R. (1992). In vitro model of essential fatty acid deficiency. J. Investig. Dermatol..

[24] Van Smeden J., Boiten W.A., Hankemeier T., Rissmann R., Bouwstra J.A., Vreeken R.J. (2014). Combined LC/MS-platform for analysis of all major stratum corneum lipids, and the profiling of skin substitutes. Biochim. Biophys. Acta.

[25] Duque-Fernandez A., Gauthier L., Simard M., Jean J., Gendreau I., Morin A., Soucy J., Auger M., Pouliot R. (2016). A 3D-psoriatic skin model for dermatological testing: The impact of culture conditions. Biochem. Biophys. Rep..

[26] Netzlaff F., Kaca M., Bock U., Haltner-Ukomadu E., Meiers P., Lehr C.M., Schaefer U.F. (2007). Permeability of the reconstructed human epidermis model Episkin in comparison to various human skin preparations. Eur. J. Pharm. Biopharm..

[27] Sun R., Celli A., Crumrine D., Hupe M., Adame L., Pennypacker S., Park K., Uchida Y., Feingold K.R., Elias P.M. (2014). Functional Epidermal Permeability Barrier in Human Epidermal Equivalents. Tissue Eng..

[28] Marcelo C.L., Rhodes L.M., Dunham W.R. (1994). Normalization of essential-fatty-acid-deficient keratinocytes requires palmitic acid. J. Investig. Dermatol..

[29] Garner W.L., Oyatsu Y., Zuccaro C., Rodriquez J.L., Smith D.J., Marcelo C.L. (1995). The effect of essential fatty acid supplementation on keratinocyte replication. Prostaglandins Leukot. Essent. Fat. Acids.

[30] Breiden B., Gallala H., Doering T., Sandhoff K. (2007). Optimization of submerged keratinocyte cultures for the synthesis of barrier ceramides. Eur. J. Cell Biol..

[31] Vicanova J., Weerheim A.M., Kempenaar J.A., Ponec M. (1999). Incorporation of linoleic acid by cultured human keratinocytes. Arch. Dermatol. Res..

[32] Boyce S.T., Williams M.L. (1993). Lipid supplemented medium induces lamellar bodies and precursors of barrier lipids in cultured analogues of human skin. J. Investig. Dermatol..

[33] Thakoersing V.S., Smeden J., Boiten W.A., Gooris G.S., Mulder A.A., Vreeken R.J., El Ghalbzouri A., Bouwstra J.A. (2015). Modulation of stratum corneum lipid composition and organization of human skin equivalents by specific medium supplements. Exp. Dermatol..

[34] Fujii M., Ohyanagi C., Kawaguchi N., Matsuda H., Miyamoto Y., Ohya S., Nabe T. (2018). Eicosapentaenoic acid ethyl ester ameliorates atopic dermatitis-like symptoms in special diet-fed hairless mice, partly by restoring covalently bound ceramides in the stratum corneum. Exp. Dermatol..

[35] Germain L., Rouabhia M., Guignard R., Carrier L., Bouvard V., Auger F.A. (1993). Improvement of human keratinocyte isolation and culture using thermolysin. Burns.

[36] Jean J., Lapointe M., Soucy J., Pouliot R. (2009). Development of an in vitro psoriatic skin model by tissue engineering. J. Dermatol. Sci..

[37] Pouliot R., Larouche D., Auger F.A., Juhasz J., Xu W., Li H., Germain L. (2002). Reconstructed human skin produced in vitro and grafted on athymic mice. Transplantation.

[38] Franz T.J. (1975). Percutaneous absorption on the relevance of in vitro data. J. Investig. Dermatol..

[39] Michel M., Auger F.A., Germain L. (1993). Anchored skin equivalent cultured in vitro: A new tool for percutaneous absorption studies. In Vitro Cell. Dev. Biol. Anim..

[40] Julien C., Berthiaume L., Hadj-Tahar A., Rajput A.H., Bedard P.J., Di Paolo T., Julien P., Calon F. (2006). Postmortem brain fatty acid profile of levodopa-treated Parkinson disease patients and parkinsonian monkeys. Neurochem. Int..

[41] Gevariya N., Besancon M., Robitaille K., Picard V., Diabate L., Alesawi A., Julien P., Fradet Y., Bergeron A., Fradet V. (2019). Omega-3 fatty acids decrease prostate cancer progression associated with an anti-tumor immune response in eugonadal and castrated mice. Prostate.

[42] Yago M.D., Diaz R.J., Ramirez R., Martinez M.A., Manas M., Martinez-Victoria E. (2004). Dietary-induced changes in the fatty acid profile of rat pancreatic membranes are associated with modifications in acinar cell function and signalling. Br. J. Nutr..

[43] Elias P.M., Brown B.E., Ziboh V.A. (1980). The permeability barrier in essential fatty acid deficiency: Evidence for a direct role for linoleic acid in barrier function. J. Investig. Dermatol..

[44] Wertz P.W., Cho E.S., Downing D.T. (1983). Effect of essential fatty acid deficiency on the epidermal sphingolipids of the rat. Biochim. Biophys. Acta.

[45] Hansen H.S., Jensen B. (1985). Essential function of linoleic acid esterified in acylglucosylceramide and acylceramide in maintaining the epidermal water permeability barrier. Evidence from feeding studies with oleate, linoleate, arachidonate, columbinate and alpha-linolenate. Biochim. Biophys. Acta.

[46] Fujii M., Nakashima H., Tomozawa J., Shimazaki Y., Ohyanagi C., Kawaguchi N., Ohya S., Kohno S., Nabe T. (2013). Deficiency of n-6 polyunsaturated fatty acids is mainly responsible for atopic dermatitis-like pruritic skin inflammation in special diet-fed hairless mice. Exp. Dermatol..

[47] Van de Sandt J.J., van Burgsteden J.A., Cage S., Carmichael P.L., Dick I., Kenyon S., Korinth G., Larese F., Limasset J.C., Maas W.J. (2004). In vitro predictions of skin absorption of caffeine, testosterone, and benzoic acid: A multi-centre comparison study. Regul. Toxicol. Pharmacol..

[48] Schreiber S., Mahmoud A., Vuia A., Rubbelke M.K., Schmidt E., Schaller M., Kandarova H., Haberland A., Schafer U.F., Bock U. (2005). Reconstructed epidermis versus human and animal skin in skin absorption studies. Toxicol. In Vitro.

[49] Michel M., Germain L., Belanger P.M., Auger F.A. (1995). Functional evaluation of anchored skin equivalent cultured in vitro: Percutaneous absorption studies and lipid analysis. Pharm. Res..

[50] Barry B.W. (1991). Lipid-Protein-Partitioning theory of skin penetration enhancement. J. Control. Release.

[51] Wilkinson S.C., Maas W.J., Nielsen J.B., Greaves L.C., van de Sandt J.J., Williams F.M. (2006). Interactions of skin thickness and physicochemical properties of test compounds in percutaneous penetration studies. Int. Arch. Occup. Environ. Health.

[52] Holub B.J. (2002). Clinical nutrition: 4. Omega-3 fatty acids in cardiovascular care. CMAJ.

[53] Simopoulos A.P. (2002). The importance of the ratio of omega-6/omega-3 essential fatty acids. Biomed. Pharm..

[54] Pawlosky R.J., Hibbeln J.R., Lin Y., Goodson S., Riggs P., Sebring N., Brown G.L., Salem N. (2003). Effects of beef- and fish-based diets on the kinetics of n-3 fatty acid metabolism in human subjects. Am. J. Clin. Nutr..

[55] Arterburn L.M., Hall E.B., Oken H. (2006). Distribution, interconversion, and dose response of n-3 fatty acids in humans. Am. J. Clin. Nutr..

[56] Drouin G., Rioux V., Legrand P. (2019). The n-3 docosapentaenoic acid (DPA): A new player in the n-3 long chain polyunsaturated fatty acid family. Biochimie.

[57] Calder P.C. (2012). Long-chain fatty acids and inflammation. Proc. Nutr. Soc..

[58] Lu L., Okada N., Nakatani S., Yoshikawa K. (1995). Eicosapentaenoic acid-induced changes in membrane fluidity and cell adhesion molecules in cultured human keratinocytes. Br. J. Dermatol..

[59] Elias P.M. (2012). Structure and function of the stratum corneum extracellular matrix. J. Investig. Dermatol..

[60] Candi E., Schmidt R., Melino G. (2005). The cornified envelope: A model of cell death in the skin. Nat. Rev. Mol. Cell Biol.

[61] Kew S., Banerjee T., Minihane A.M., Finnegan Y.E., Williams C.M., Calder P.C. (2003). Relation between the fatty acid composition of peripheral blood mononuclear cells and measures of immune cell function in healthy, free-living subjects aged 25–72 y. Am. J. Clin. Nutr..

[62] Hanley K., Jiang Y., He S.S., Friedman M., Elias P.M., Bikle D.D., Williams M.L., Feingold K.R. (1998). Keratinocyte differentiation is stimulated by activators of the nuclear hormone receptor PPARalpha. J. Investig. Dermatol..

[63] Chon S.H., Tannahill R., Yao X., Southall M.D., Pappas A. (2015). Keratinocyte differentiation and upregulation of ceramide synthesis induced by an oat lipid extract via the activation of PPAR pathways. Exp. Dermatol..

[64] Batheja P., Song Y., Wertz P., Michniak-Kohn B. (2009). Effects of growth conditions on the barrier properties of a human skin equivalent. Pharm. Res..

[65] Schmuth M., Moosbrugger-Martinz V., Blunder S., Dubrac S. (2014). Role of PPAR, LXR, and PXR in epidermal homeostasis and inflammation. Biochim. Biophys. Acta.

[66] Wijesinghe D.S., Brentnall M., Mietla J.A., Hoeferlin L.A., Diegelmann R.F., Boise L.H., Chalfant C.E. (2014). Ceramide kinase is required for a normal eicosanoid response and the subsequent orderly migration of fibroblasts. J. Lipid Res..

