# Supplementary material for: Modulation of the Lipid Profile of Reconstructed Skin Substitutes after Essential Fatty Acid Supplementation Affects Testosterone Permeability
Source: Cells. 2019 Sep 25;8(10):1142. doi: 10.3390/cells8101142 (PMC6829228; doi:10.3390/cells8101142)
Supplement: Supplementary file 1 [file cells-08-01142-s001.pdf]

SUPPLEMENTARY MATERIAL

Table S1. Levels of phospholipid fatty acids in epidermis and dermis of skin substitutes after EFA supplementation.

Fatty acids	Epidermis						Dermis					
	Mean ±SD (μg/g of tissue)		P-value	Mean ±SD (μg/g of tissue)		P-value	Mean ±SD (μg/g of tissue)		P-value	Mean ±SD (μg/g of tissue)		P-value
	Substitute^{-}	Substitute^{ALA+}		Substitute^{-}	Substitute^{LA+}		Substitute^{-}	Substitute^{ALA+}		Substitute^{-}	Substitute^{LA+}	
**Saturated FAs**												
14:0 (Myristic acid)	35.1 ± 12.7	35.6 ± 15.0	NS	28.7 ± 7.0	39.0 ± 3.8	NS	70.7 ± 12.0	56.8 ± 26.2	NS	71.4 ± 30.1	44.2 ± 12.1	NS
16:0 (Palmitic acid)	282.3 ± 133.5	271.3 ± 87.7	NS	186.3 ± 25.9	240.4 ± 24.4	<0.0001	449.3 ± 63.6	354.8 ± 141.4	NS	453.1 ± 139.2	334.4 ± 81.0	NS
18:0 (Stearic acid)	220.8 ± 105.2	226.9 ± 71.1	NS	151.4 ± 19.9	198.0 ± 27.8	<0.0001	424.3 ± 87.1	381.2 ± 142.5	NS	401.2 ± 110.6	335.1 ± 55.2	NS
**n-3 PUFAs**												
18:3n-3 (ALA)	0 ± 0	3.5 ± 5.9	NS	0 ± 0	0 ± 0	NS	0 ± 0	22.5 ± 14.0	NS	0.6 ± 1.4	0 ± 0	NS
20:5n-3 (EPA)	2.5 ± 2.8	30.3 ± 13.5	<0.0001	0.38 ± 0.86	1.8 ± 1.4	NS	0 ± 0	55.6 ± 31.0	<0.0001	3.9 ± 4.9	0 ± 0	NS
22:5n-3 (DPA)	9.2 ± 7.2	34.5 ± 11.7	<0.0001	6.7 ± 1.2	7.5 ± 0.7	NS	42.3 ± 11.8	123.9 ± 34.2	<0.0001	32.3 ± 9.8	23.1 ± 6.0	NS
22:6n-3 (DHA)	10.3 ± 7.8	13.9 ± 4.8	NS	7.8 ± 1.8	8.4 ± 1.8	NS	50.6 ± 21.1	42.0 ± 9.4	NS	40.7 ± 16.1	28.7 ± 6.9	0.011
**n-6 PUFAs**												
18:2n-6 (LA)	116.3 ± 74.0	114.8 ± 65.7	NS	69.7 ± 23.3	105.7 ± 12.8	<0.0001	100.8 ± 40.6	152.5 ± 77.01	NS	131.4 ± 37.4	200.2 ± 68.8	NS
18:3n-6 (γ-linolenic acid)	0 ± 0	0 ± 0	NS	1.5 ± 3.4	1.6 ± 3.9	NS	1.1 ± 1.8	0.5 ± 0.77	NS	0.6 ± 1.5	1.6 ± 1.8	NS
20:3n-6 (dihomo-γ-linolenic acid)	15.2 ± 11.9	12.5 ± 6.7	NS	12.2 ± 2.6	18.4 ± 3.6	NS	15.5 ± 2.1	16.8 ± 7.65	NS	20.4 ± 6.6	16.7 ± 6.2	NS
20:4n-6 (AA)	50.8 ± 41.7	39.0 ± 13.6	NS	32.5 ± 6.5	51.5 ± 8.4	0.0031	418.7 ± 111.1	251.2 ± 87.7	<0.0001	348.1 ± 175.2	334.7 ± 79.4	NS
**n-7 MUFAs**												
16:1n-7 (Palmitoleic acid)	157.2 ± 87.8	163.1 ± 95.8	NS	120.9 ± 31.6	158.0 ± 19.8	NS	66.7 ± 3.9	60.9 ± 27.9	NS	131.1 ± 135.1	42.9 ± 10.9	NS
18:1n-7 (Vaccenic acid)	252.1 ± 132.0	199.5 ± 66.9	NS	191.7 ± 30.0	226.3 ± 31.5	NS	214.4 ± 58.5	120.3 ± 44.3	0.0232	266.1 ± 171.6	108.2 ± 20.8	0.043
**n-9 MUFA**												
18:1n-9 (Oleic acid)	594.8 ± 347.7	561.1 ± 252.4	NS	398.2 ± 71.7	491.7 ± 53.2	<0.0001	492.8 ± 102.6	381.3 ± 135.9	NS	601.2 ± 315.7	300.6 ± 55.8	0.0002
**Totals**												
Total PUFAs	219.0 ± 152.8	259.7 ± 121.9	NS	147.2 ± 46.4	220.1 ± 25.3	NS	796.0 ± 195.1	728.9 ± 271.0	NS	731.0 ± 223.1	750.6 ± 183.0	NS
Total MUFAs	1054.3±580.7	955.8±416.2	NS	741.4±132.7	911.9±106.9	NS	892.0±187.9	625.8±219.1	NS	1093.4±617.0	519.7±80.5	0.01
Total FAs	1913.7±1021	1895.9±764.9	NS	1347.0±230.3	1719.0±160.1	0.0116	2813.2±604.4	2232.2±825.4	NS	2818.0±890.8	2088.5±439.6	NS

Abbreviations : AA, arachidonic acid; ALA, α-linolenic acid; DHA, docosahexaenoic acid; EPA, eicosapentaenoic acid; FA, fatty acid; LA, linoleic acid; MUFA, monounsaturated fatty acid; PUFAs, polyunsaturated fatty acids; SFA, saturated fatty acid; SD, standard deviation.

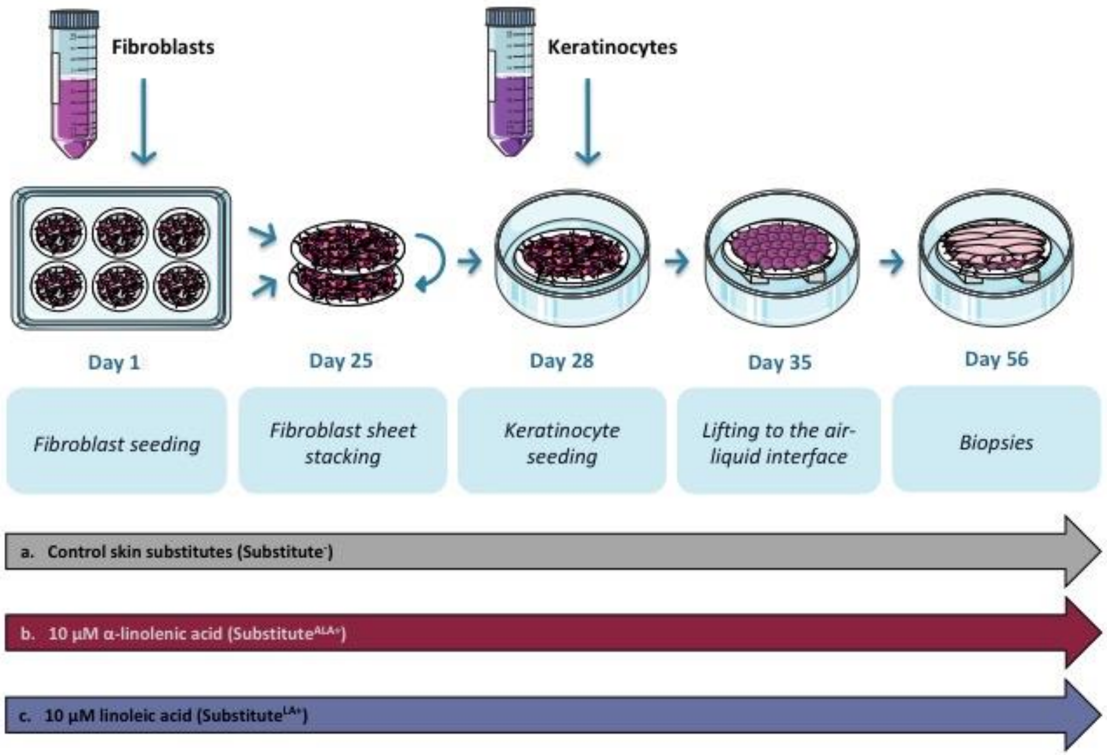

Figure S1. Schematic overview of skin substitute reconstruction by the self-assembly method. (Day 1) Fibroblasts were cultured in 6-well plates to form manipulatable sheets. (Day 25) Two sheets were superimposed in a 100 mm Petri dish and incubated for three days to form the dermal layer. (Day 28) Keratinocytes were seeded on the dermal equivalent to form a new epidermal layer and after seven days of culture (Day 35) the substitutes were raised to the air-liquid interface for 21 days. After a total of 56 days of culture, biopsies of the skin substitutes were taken and analyzed.

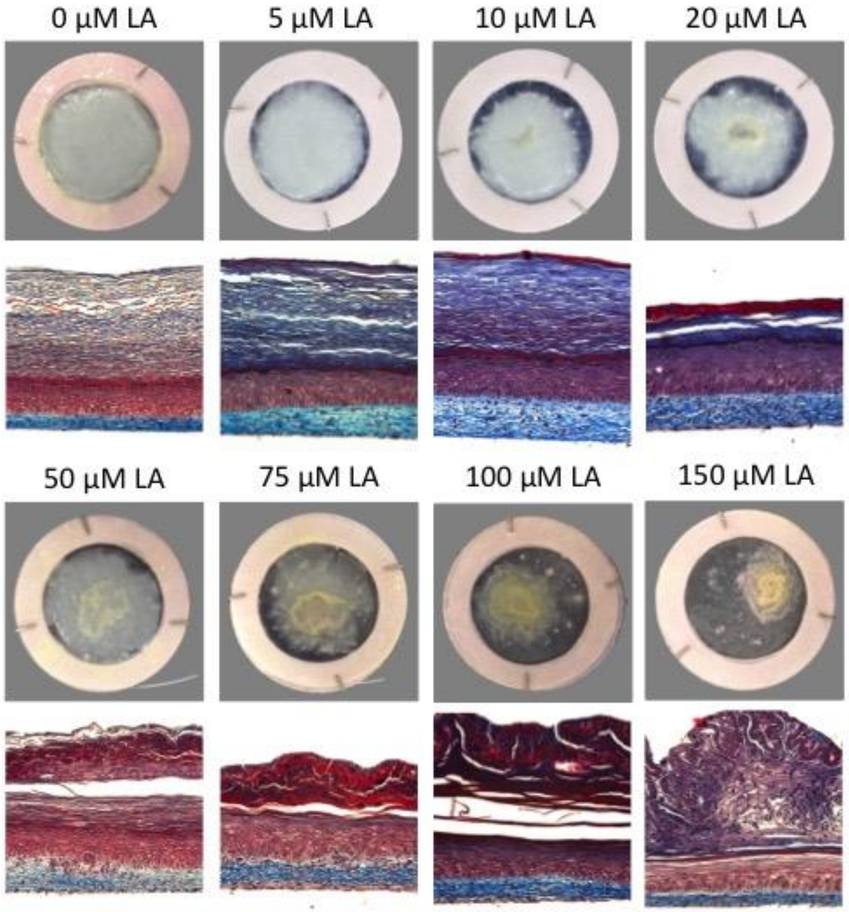

Figure S2. Effects of LA on the morphology of the skin substitutes produced using a range of concentrations (5 μM, 10 μM, 20 μM, 50 μM, 75 μM, 100 μM and 150 μM).

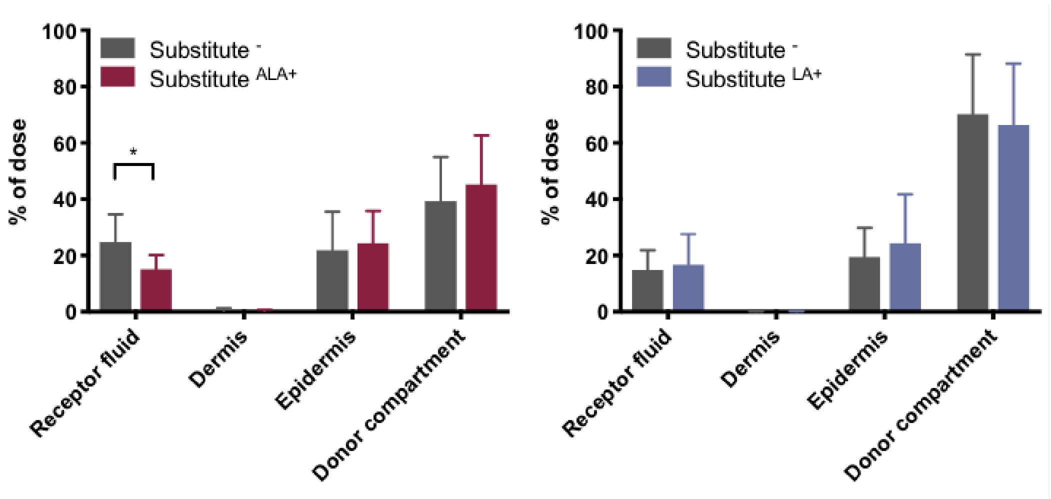

Figure S3. *In vitro* percutaneous absorption of testosterone through skin substitutes, expressed as percentage of the dose recovered in the different compartments after 24 h. Values are mean +/- SD (3 donors, 6 skin substitutes per donor), Two-way ANOVA followed by Tukey's *post-hoc* test. *P<0.05.

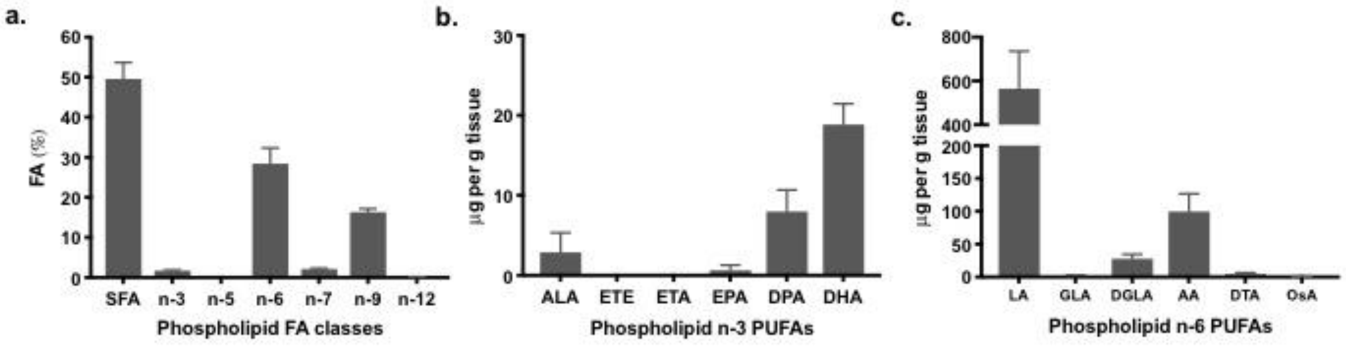

Figure S4. Human skin lipid profile. (**a**) Proportion of different FA classes in phospholipids of human epidermis. (**b**) n-3 PUFAs and (**c**) n-6 PUFAs in phospholipids of human epidermis. Values are means ± SD (n=6). Abbreviations: AA, arachidonic acid; ALA, α-linolenic acid; DGLA, dihomo-γ-linolenic acid; DHA, docosahexaenoic acid; DPA, docosapentaenoic acid; DTA, docosatetraenoic acid; EPA, eicosapentaenoic acid; ETA, eicosatetraenoic acid; ETE, eicosatrienoic acid: GLA: γ-linolenic acid; LA, linoleic acid; OsA, osbond acid; PUFAs, polyunsaturated fatty acids.
